# Optimization of SPECT/CT imaging protocols for quantitative and qualitative ^99m^Tc SPECT

**DOI:** 10.1186/s40658-021-00405-3

**Published:** 2021-07-30

**Authors:** Dennis Kupitz, Heiko Wissel, Jan Wuestemann, Stephanie Bluemel, Maciej Pech, Holger Amthauer, Michael C. Kreissl, Oliver S. Grosser

**Affiliations:** 1 Department of Radiology and Nuclear Medicine, University Hospital Magdeburg, Magdeburg, Germany; 2 Department of Nuclear Medicine, Charité - Universitätsmedizin Berlin, Corporate Member of Freie Universität Berlin, Humboldt-Universität zu Berlin, and Berlin Institute of Health, Berlin, Germany; 3 Research Campus STIMULATE, Otto-von-Guericke University, Magdeburg, Germany

**Keywords:** Quantitative SPECT, SPECT/CT, Optimization, Scatter correction, Image reconstruction

## Abstract

**Background:**

The introduction of hybrid SPECT/CT devices enables quantitative imaging in SPECT, providing a methodological setup for quantitation using SPECT tracers comparable to PET/CT. We evaluated a specific quantitative reconstruction algorithm for SPECT data using a ^99m^Tc-filled NEMA phantom. Quantitative and qualitative image parameters were evaluated for different parametrizations of the acquisition and reconstruction protocol to identify an optimized quantitative protocol.

**Results:**

The reconstructed activity concentration (AC_rec_) and the signal-to-noise ratio (SNR) of all examined protocols (*n* = 16) were significantly affected by the parametrization of the weighting factor *k* used in scatter correction, the total number of iterations and the sphere volume (all, *p* < 0.0001). The two examined SPECT acquisition protocols (with 60 or 120 projections) had a minor impact on the AC_rec_ and no significant impact on the SNR.

In comparison to the known AC, the use of default scatter correction (*k* = 0.47) or object-specific scatter correction (*k* = 0.18) resulted in an underestimation of AC_rec_ in the largest sphere volume (26.5 ml) by − 13.9 kBq/ml (− 16.3%) and − 7.1 kBq/ml (− 8.4%), respectively. An increase in total iterations leads to an increase in estimated AC and a decrease in SNR. The mean difference between AC_rec_ and known AC decreased with an increasing number of total iterations (e.g., for 20 iterations (2 iterations/10 subsets) = − 14.6 kBq/ml (− 17.1%), 240 iterations (24i/10s) = − 8.0 kBq/ml (− 9.4%), *p* < 0.0001). In parallel, the mean SNR decreased significantly from 2i/10s to 24i/10s by 76% (*p* < 0.0001).

**Conclusion:**

Quantitative SPECT imaging is feasible with the used reconstruction algorithm and hybrid SPECT/CT, and its consistent implementation in diagnostics may provide perspectives for quantification in routine clinical practice (e.g., assessment of bone metabolism). When combining quantitative analysis and diagnostic imaging, we recommend using two different reconstruction protocols with task-specific optimized setups (quantitative vs. qualitative reconstruction). Furthermore, individual scatter correction significantly improves both quantitative and qualitative results.

**Supplementary Information:**

The online version contains supplementary material available at 10.1186/s40658-021-00405-3.

## Background

During the last decade, quantitative imaging has become a standard in nuclear medicine diagnostics. This benchmark was exemplified by the long-term process in developing PET/CT (positron emission tomography with X-ray computed tomography), representing the current standard in quantitative hybrid imaging [1, 2].

In parallel, the introduction of hybrid SPECT/CT (single photon emission computed tomography with computed X-ray tomography) imaging devices provides opportunities for quantitation in SPECT imaging [3]. In this context, quantitative SPECT (qSPECT) provides the possibility to use gamma-emitting radiotracers within a similar setup as PET/CT applications. Apart from superior resolution in PET, this development has to overcome the substantial difference in quantitation between PET/CT and SPECT/CT. Currently, novel clinical applications (e.g., dosimetry in peptide receptor radionuclide therapy) further emphasize the need for the development of qSPECT imaging [4–6].

As a result of improvements in SPECT image reconstruction, dedicated corrections (e.g., attenuation correction and scatter correction) have provided the potential for qSPECT imaging [4, 7–13]. Currently, state-of-the-art attenuation correction in SPECT reconstruction is performed using CT data from hybrid SPECT/CT imaging [13, 14]. Various methods have been implemented for scatter correction [15]. The most commonly used method is based on the parallel acquisition of at least one additional energy window in correlation with the photopeak window (or to each photopeak window, if applicable) [16–19].

In this study, we evaluated a manufacturer-specific quantitative reconstruction algorithm for SPECT imaging using ^99m^Tc (Technetium-99m)-labelled radiopharmaceuticals. We assessed the accuracy in quantitation of activity concentrations for different parametrizations of the acquisition and reconstruction protocol (e.g., iterations and scatter weighting) to identify an optimized quantitative protocol. In parallel, qualitative image parameters (e.g., signal-to-noise ratio) were measured and evaluated. The phantom setup used for evaluation was adapted to clinical conditions (e.g., observed activity concentrations).

## Methods

### Phantom

Phantom measurements were performed using the standardized NEMA IEC body phantom (Data Spectrum Corporation, Durham, NC, USA) with six fillable spheres (diameters of 10, 13, 17, 22, 28 and 37 mm) and a cylindrical polystyrene-filled lung insert [20]. The phantom was filled with ^99m^Tc-pertechnetate diluted in water, with specific activities of 80 kBq/ml for each of the six spheres and 10 kBq/ml for the background (ratio 8:1). The lung insert, in the centre of the phantom, was not filled with radioactive material.

### SPECT/CT: imaging and reconstruction

Imaging was performed with hybrid SPECT/CT (Discovery NM/CT 670, GE Healthcare, Haifa, Israel) with dual-head NaI detectors equipped with low-energy, high-resolution (LEHR) collimators. SPECT data were acquired using two different acquisition protocols: (1.) a clinical ^99m^Tc imaging protocol with 60 projections per 360° (30 projections per detector) at steps of 6° (20 s/projection) and (2.) a NEMA-oriented imaging protocol [21] with 120 projections per 360° (60 per detector) at steps of 3° (10 s/projection to achieve identic scan time and comparable total count statistics as setup number 1). The second protocol was chosen to analyse the effect by number of projections for equivalent total examination time. All other imaging parameters (energy window: photopeak = 140.5 keV ± 10%, scatter window = 120.0 keV ± 5%; image matrix 256 × 256, pixel size = 2.21 × 2.21 mm, and zoom = 1.0) were not changed.

CT imaging was performed using a low-dose protocol (*I* = 40 mA, *U* = 120 kVp, *t*_rot_ = 0.5 s, primary collimation = 16 × 1.25 mm, and pitch = 1.375). CT data were reconstructed with a matrix of 512 × 512 (pixel size = 0.98 × 0.98 mm) and a slice thickness of 3.75 mm by filtered back projection (with manufacturer-specific convolution kernel “standard”). The low-dose CT data were used for attenuation correction of the corresponding SPECT data.

Quantitative SPECT reconstruction was performed using a dedicated software module (Q. Metrix, GE Healthcare, Haifa, Israel) [22] used an iterative ordered-subset expectation maximization algorithm (2D OSEM) for image reconstruction with four different parametrizations: (1.) 2 iterations and 10 subsets (2i/10s), (2.) 4 iterations and 10 subsets (4i/10s), (3.) 5 iterations and 15 subsets (5i/15s) and (4.) 24 iterations and 10 subsets (24i/10s). The reconstruction algorithm performed corrections for resolution recovery (by using a collimator- and nuclide-specific 3D-point spread function), attenuation and scatter. The dual-energy window method was used for scatter correction [10]. SPECT data were reconstructed in general without postfiltering. For comparison, SPECT data reconstructions with 2i/10s were performed with postfiltering (Butterworth with cut-off frequency = 0.5 cycles/cm and power = 10) to illustrate the image quality of our local clinical standard for diagnostic ^99m^Tc imaging.

Analysis was performed for two different scatter weighting factors (SCFs): SCF = 1.10 (SCF_1.10_, default for ^99m^Tc, recommended by the manufacturer) and SCF = 0.41 (SCF_0.41_, optimized for chosen phantom geometry). The optimized SCF_0.41_ was previously determined in concordance with de Nijs et al. [23] (see supplementary information). For the used energy window widths, the two SCFs can be converted into the more common scatter multipliers (introduced by Jaszczak [16]) *k* = 0.470 and 0.175 for SCF_1.10_ and SCF_0.41_, respectively [10, 24]. Table 1 shows an overview of all analysed reconstructions.

**Table 1:** Examined reconstruction protocols (*n* = 16)

	Iterations and subsets	SCF
Quantitative reconstructions^a^		
Clinical acquisition protocol^b^	4i/10s	0.41 and 1.10
	5i/15s	0.41 and 1.10
	24i/10s	0.41 and 1.10
NEMA acquisition protocol^c^	4i/10s	0.41 and 1.10
	5i/15s	0.41 and 1.10
	24i/10s	0.41 and 1.10
Diagnostic reconstructions^d^		
Clinical acquisition protocol^b^	2i/10s^e^	0.41 and 1.10^e^
NEMA acquisition protocol^c^	2i/10s	0.41 and 1.10

^a^Without postfiltering,

^b^Sixty projections with 20 s/projection,

^c^One hundred twenty projections with 10 s/projection,

^d^With postfiltering (Butterworth, cut-off frequency = 0.5 cycles/cm, power = 10)

^e^Institutional standard for diagnostic imaging, 2i/10s and SCF = 1.10

*SCF* scatter weighting factor

The conversion from reconstructed counts into activity concentration (qSPECT data in units of MBq/ml) was performed by multiplication with a quantitative factor QF (MBq/cnt/ml) [22, 25]:

1 $$ \mathrm{QF}=\frac{1}{S\bullet T\bullet {V}_{\mathrm{Voxel}}} $$

The effective scanning time of the acquisition was represented by *T* (*T* = 1200 s, constant for all examined acquisition protocols). *V*_voxel_ represents the volume of a single reconstructed voxel (*V*_voxel_ = 0.01079 ml). The planar system sensitivity *S* of the gamma camera (units of cnt/s/MBq) was determined in concordance with the NEMA NU 1-2018 formalism [21].

### Volume of interest analysis

The software PMOD (PMOD Ver. 3.805, PMOD Technologies LLC, Zurich, Switzerland) was used for segmentation of spherical volumes of interest (VOIs) in the low-dose CT data by specifying the known diameter of the spheres. Subsequently, these VOIs were transferred to the co-registered reconstructed SPECT. VOIs were defined for all six spheres of the IEC phantom and for a spherical background VOI (*V* = 49.9 ml). For the evaluation of the reconstructed voxel values per sphere, the reconstructed counts were converted into AC values by using equation (1). The AC values of all voxels per sphere were considered in the analysis. The smallest spherical VOI (*V* = 0.5 ml) consists of *n* = 46 voxels and the largest spherical VOI (*V* = 26.5 ml) of *n* = 2438 voxels. The background VOI contains *n* = 4612 voxels.

Recovery coefficients (hot spot recovery coefficients (HSRC)) [26] were determined using the mean AC (AC_rec_) in the spheres estimated from the qSPECT data (AC_rec.sphere_) and the known activity concentration (AC_real.sphere_):

2 $$ \mathrm{HSRC}=\frac{{\mathrm{AC}}_{\mathrm{rec}.\mathrm{sphere}}}{{\mathrm{AC}}_{\mathrm{real}.\mathrm{sphere}}} $$

The signal-to-noise ratio (SNR) was calculated as the difference of the means of the reconstructed AC in the sphere and the background in relation to the standard deviation (SD) of the background VOI SD_rec. BG_ [27]:

3 $$ \mathrm{SNR}=\frac{\left({\mathrm{AC}}_{\mathrm{rec}.\mathrm{sphere}}-{\mathrm{AC}}_{\mathrm{rec}.\mathrm{BG}}\right)}{{\mathrm{SD}}_{\mathrm{rec}.\mathrm{BG}}} $$

The SPECT image noise (*N*) was determined within the background by the ratio of SD of the background VOI and the mean reconstructed AC of the background [27]:

4 $$ N\left[\%\right]=\frac{{\mathrm{SD}}_{\mathrm{rec}.\mathrm{BG}}}{{\mathrm{AC}}_{\mathrm{rec}.\mathrm{BG}}}\bullet 100\% $$

### Statistics

The R software package (version 3.3.3, R Foundation for Statistical Computing, Vienna, Austria) was used for statistical evaluations of the reconstructed AC values. Levene’s test and the Shapiro-Wilk test were used to check the equality of variances and normal distribution of the variables, respectively. Differences in nonparametric, dependent variables were tested for significance by using the Friedman test or Wilcoxon signed-rank test, if applicable. ANOVA or the *t* test with Bonferroni–Holm correction applied for multiple comparisons was used for parametrically distributed dependent variables. All tests were performed two-sided, and significance was assumed at a *p* value < 0.05.

## Results

The planar sensitivities for ^99m^Tc were estimated for detector 1 S_DET1_ and detector 2 S_DET2_ to be 71.7 cps/MBq and 72.8 cps/MBq, respectively. For methodological limitations, a mean system sensitivity of *S*_system_ = 72.3 cps/MBq was used. The known AC in the spheres was 85.1 kBq/ml and 10.6 kBq/ml in the background (ratio ≈ 8:1).

AC_rec_, HSRC and SNR were not normally distributed when comparing the analysed structures (VOIs) in the phantom for each examined reconstruction protocol (Table 1). In contrast, AC_rec_, HSRC, SNR and Noise were normally distributed for each VOI (e.g., comparing only the largest sphere VOIs or only the background VOIs).

### Quantitative results

#### Spheres

The AC_rec_ and HSRCs were significantly affected by the sphere volume and by the reconstruction protocol (all *p* < 0.0001, result by the Friedman test).

The HSRC depended significantly on sphere volume (*p* < 0.0001). The effect by volume will be exemplified for the two largest spheres: the HSRC of the second largest sphere (*V* = 11.5 ml, HSRC = 0.85) was significantly reduced by − 0.03 (representing ΔAC_rec_ = − 2.7 kBq/ml, *p* = 0.01) compared with the largest sphere (*V* = 26.5 ml, HSRC = 0.88).

The results from ANOVA analysis showed significant dependencies of the HSRC and the AC_rec_ on the number of total iterations (all, *p* < 0.0001), SCF (all, *p* < 0.0001) and acquisition protocol (all, *p* < 0.02) for each of the four largest examined sphere volumes (up to *V* = 2.6 ml). Table 2 shows the results of an ANOVA post hoc test for the largest examined sphere. The results of the remaining sphere volumes are provided in the supplementary data (supplementary Table 1).

**Table 2:** Effect of examined parameters on AC_rec_, HSRC and SNR. The results are exemplified for the largest sphere volume in comparison to a reference protocol^a^.

Parameter of variation	ΔAC_rec_	*p*^b^	ΔHSRC	*p*^b^	ΔSNR	*p*^b^
	(kBq/ml)					
SCF: 0.41	+ 6.7	< 0.0001	+ 0.08	< 0.0001	+ 1.1	n.s.^d^
Iteration set: 4i/10s	+ 4.1	< 0.0001	+ 0.05	< 0.0001	− 8.2	< 0.0001
Iteration set: 5i/15s	+ 5.6	< 0.0001	+ 0.07	< 0.0001	− 11.9	< 0.0001
Iteration set: 24i/10s	+ 6.6	< 0.0001	+ 0.08	< 0.0001	− 17.9	< 0.0001
Acquisition: NEMA^c^	− 0.9	0.04	− 0.01	0.04	+ 0.6	n.s.^d^

Analysis performed for the largest sphere. For residual spheres see supplementary Table 1.

^a^Clinical standard for diagnostic imaging and reconstruction (60 projections with 20 s/projection, SCF_1.10_, 2i/10s, Butterworth postfilter)

^b^ANOVA post hoc test

^c^NEMA acquisition protocol (120 projections and 10 s/projection)

^d^Not significant, tested by ANOVA

*SCF* scatter weighting factor, *AC*_*rec*_ mean reconstructed activity concentration, *HSRC* hot spot recovery coefficient, *SNR* signal-to-noise ratio

**Fig. 1:**
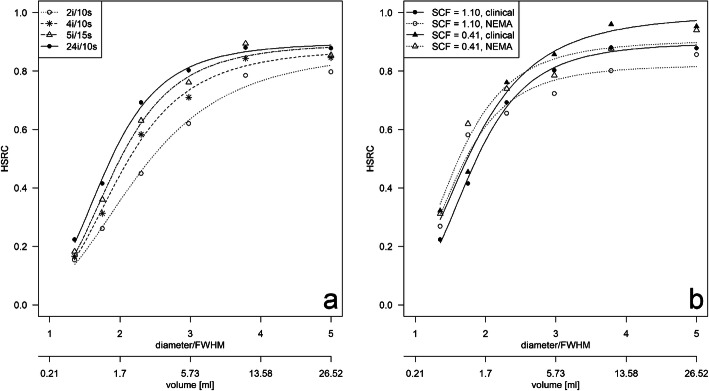
Effect of the reconstruction setup on the hot spot recovery coefficients (HSRC) of the examined spheres. **a** HSRCs of the clinical acquisition protocol (60 projections, 20 s/projection) for different reconstruction setups (SCF_1.10_ in combination with four different iteration sets). **b** HSRCs for a fixed set of iterations (24i/10s) demonstrating effects by the acquisition protocol (clinical or NEMA) and SCF (SCF = 1.10 or 0.41). HSRC was plotted against sphere diameter normalized to FWHM at 100 mm with LEHR collimator (FWHM = 7.4 mm) and sphere volume

The effect of the number of total iterations and the sphere volume on the HSRC is exemplified for the clinical acquisition protocol with SCF_1.10_ (manufacturer parametrization) in Fig. 1a. The HSRC curves of the four sets of iterations were significantly different from each other (*p* = 0.0007), and there was an increase in the HSRCs with increasing VOI volumes and with an increasing number of iterations. For example, the median HSRC increased in the specific setup by 0.68 (ΔAC_rec_ = 57.5 kBq/ml) when comparing the smallest sphere (*V* = 0.5 ml, AC_rec_= 14.8 kBq/ml) and the largest sphere (*V* = 26.5 ml, AC_rec_= 72.3 kBq/ml). Increasing the total number of iterations from 2i/10s to 4i/10s showed a significant increase in median HSRCs by 0.06 over all spheres (ΔAC_rec_ = 4.9 kBq/ml, *p =* 0.03). Furthermore, median HSRC increased significantly from 2i/10s to 5i/15s and from 2i/10s to 24i/10s by 0.10 (ΔAC_rec_ = 8.9 kBq/ml, *p =* 0.03) and 0.13 (ΔAC_rec_ = 11.2 kBq/ml, *p* = 0.03), respectively.

For a fixed set of total iterations (e.g., 24i/10s), the median HSRCs of the spheres increase for reconstructions with SCF_0.41_ in comparison to using a SCF_1.10_ significantly by 0.07 (ΔAC_rec_ = 5.9 kBq/ml) for the clinical and by 0.06 (ΔAC_rec_ = 5.4 kBq/ml) for the NEMA (both, *p* = 0.03) acquisition protocol, respectively. Using the clinical acquisition protocol, the median HSRCs of the spheres increased slightly by 0.02 (ΔAC_rec_ = 1.5 kBq/ml, *p* = 0.84) and by 0.02 (ΔAC_rec_ = 1.4 kBq/ml, *p* = 0.44) for SCF_1.10_ and SCF_0.41_, respectively, when compared with the NEMA acquisition protocol. The effect is exemplified by Fig. 1b.

Without recovery correction and independent of the total number of iterations, all reconstructions underestimate the known AC. For the largest examined sphere and with the phantom-specific SCF_0.41_, the AC_rec_ accuracy was in the range of − 4.8 to − 13.8% (mean = − 8.4%, − 7.1 kBq/ml; *p* < 0.0001) when compared with the known AC. All protocols with the default SCF_1.10_ underestimate the known AC in the largest sphere in the range of − 12.2 to − 22.2% (mean = − 16.3%, − 13.9 kBq/ml; *p* < 0.0001). Generally, underestimation decreases with an increase in the total number of iterations (see supplementary data, Table 2).

#### Background

The AC_rec_ of the background is higher than the known AC in the phantom for most of the examined reconstructions, and it is significantly affected by the SCF and the acquisition protocol (both, *p* < 0.0001). We did not observe a significant effect of the total number of iterations on the AC_rec_ in the background (*p* = 1). Table 3 shows the results of a post hoc test for the background.

**Table 3:** Effect of examined parameters on background AC_rec_ and *N* in comparison to a reference protocol^a^

Parameter of variation	ΔAC_rec_	*p*^b^	Δ*N*	*p*^b^
	(kBq/ml)		(%)	
SCF: 0.41	+ 1.7	< 0.0001	− 6.0	0.02
Iteration set: 4i/10s	− 0.1	n.s.^d^	+ 22.0	< 0.0001
Iteration set: 5i/15s	− 0.1	n.s.^d^	+ 35.3	< 0.0001
Iteration set: 24i/10s	− 0.2	n.s.^d^	+ 68.1	< 0.0001
Acquisition: NEMA^c^	+ 0.7	< 0.0001	− 4.1	n.s.^d^

^a^Clinical standard for diagnostic imaging and reconstruction (60 projections with 20 s/projection, SCF_1.10_, 2i/10s, Butterworth postfilter)

^b^ANOVA post hoc test

^c^NEMA acquisition protocol (120 projections and 10 s/projection)

^d^Not significant, tested by ANOVA

*SCF* scatter weighting factor, *AC*_*rec*_ mean reconstructed activity concentration, *N* noise

The smallest deviation of AC_rec_ in the background from known AC was achieved by the clinical acquisition protocol with an SCF_1.10_. These reconstructions underestimate the known AC by a mean of − 1.8%. The largest deviation from the true AC was determined for the NEMA acquisition protocol with SCF_0.41_ by a mean of 20.9%.

### Qualitative results

#### Spheres

The SNR was significantly affected by the sphere volume and by the reconstruction protocol used (both, *p* < 0.0001, results by the Friedman test).

The results from ANOVA analysis showed, for each of the four largest examined sphere volumes, a significant influence by the number of total iterations (all, *p* < 0.0001). The SCF (*p* = 0.01–0.08) and the acquisition protocol had no significant effect on the SNR (all, *p* > 0.06). Table 2 exemplifies the results of an ANOVA post hoc test for the largest examined sphere. The results of the remaining sphere volumes are provided in the supplementary data (supplementary Table 1).

**Fig. 2:**
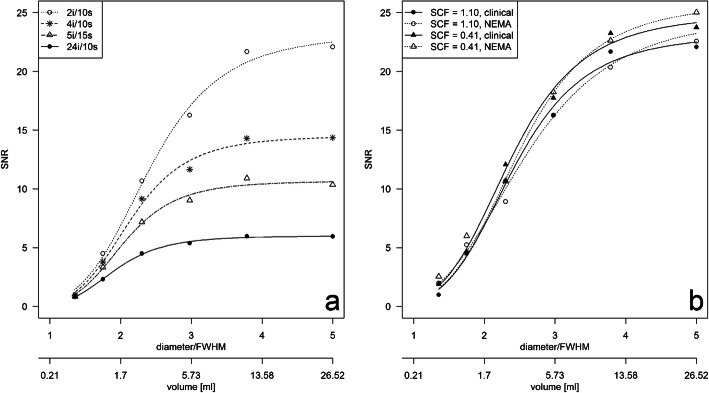
Effect of the reconstruction setup on the signal-to-noise ratio (SNR) of the examined spheres. **a** SNR of the clinical acquisition protocol (60 projections, 20 s/projection) for different reconstruction setups (SCF_1.10_ in combination with four different iteration sets). **b** SNR for a fixed set of iterations (2i/10s) demonstrating effects by the acquisition protocol (clinical or NEMA) and SCF (1.10 or 0.41). HSRC was plotted against sphere diameter normalized to FWHM at 100 mm with LEHR collimator (FWHM = 7.4 mm) and sphere volume

The effect of the total iterations and the sphere volume on the SNR was exemplified for the clinical acquisition protocol with the SCF_1.10_ (manufacturer recommendation) in Fig. 2a. The SNR in the images reconstructed by the four sets of iterations differed significantly (*p* = 0.0004), and there was an increase in the SNR with increasing VOI volumes and with a decreasing number of iterations. For example, the SNR for the smallest sphere is in the range from 0.8 (24i/10s) to 1.0 (2i/10s), while the SNR for the largest sphere varies depending on the total number of iterations from 6.0 (24i/10s) to 22.1 (2i/10s). Increasing the total number of iterations from 2i/10s to 4i/10s showed a significant decrease in median SNR by − 3.9 over all spheres (*p* = 0.03). A further increase from 2i/10s to 5i/15s or 24i/10s decreased the median SNR significantly by − 5.9 (*p* = 0.03) or by − 8.5 (*p* = 0.03), respectively.

For a fixed set of iterations, for example 2i/10s, the median SNR of the spheres increases for reconstructions with SCF_0.41_ in comparison to using an SCF_1.10_ significantly by 1.3 for the clinical and by 1.6 for the NEMA (both, *p* = 0.03) acquisition protocol. Using the clinical acquisition protocol, the median SNR of the spheres decreased slightly by − 0.2 (*p* = 0.84) and by − 0.3 (*p* = 0.69) for SCF_1.10_ and SCF_0.41_, respectively, when compared with the NEMA acquisition protocol. The effect is exemplified in Fig. 2b.

The highest SNR was observed for the diagnostic reconstruction setup (Table 1; 2i/10s with postfiltering) and SCF_0.41_ (e.g., for the largest sphere; SNR_NEMA_ = 25.0, SNR_clinical_ = 23.7). In contrast, using 24i/10s, the SNR was reduced by − 80% (SNR_NEMA_ = 5.1) and by − 74% (SNR_clinical_ = 6.2, both for the largest sphere).

#### Background

Image noise *N* in the background (Table 3) was significantly affected by the number of total iterations (*p* < 0.0001) and SCF (*p* = 0.01). The acquisition protocol had no significant effect on the SNR (*p* = 0.08).

**Fig. 3:**
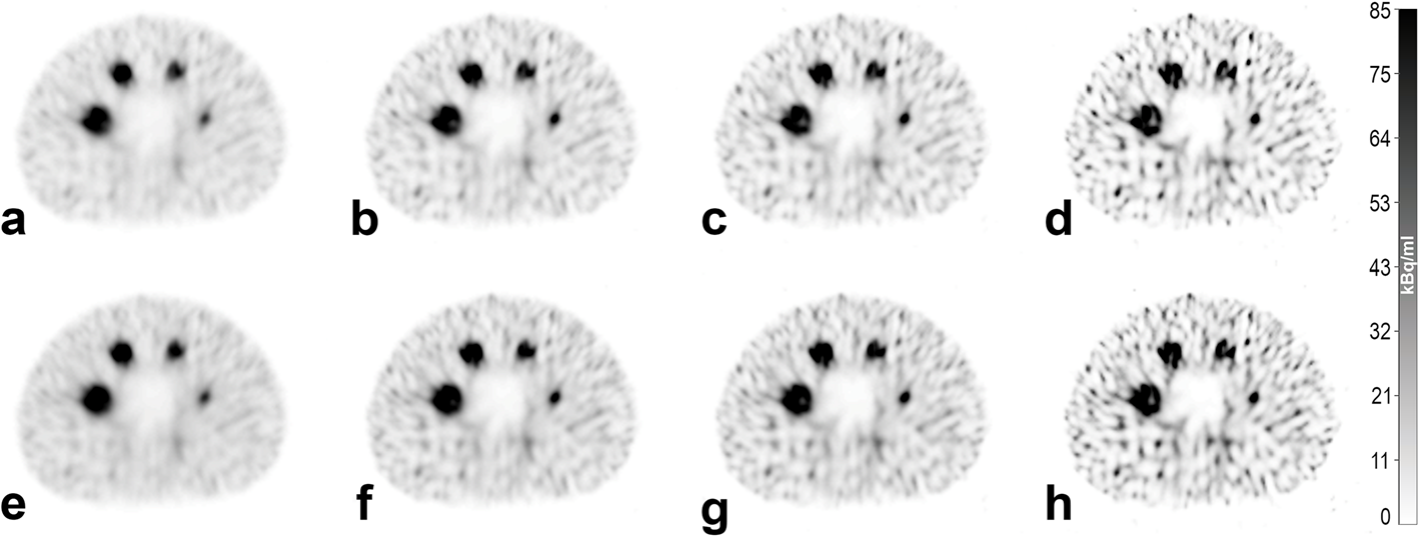
SPECT images reconstructed from data acquired with the clinical acquisition protocol. Reconstruction was performed with 2i/10s (**a** and **e**), 4i/10s (**b** and **f**), 5i/15s (**c** and **g**) and 24i/10s (**d** and **h**). Scatter correction was performed with **a**–**d** SCF = 1.10 and **e**–**h** SCF = 0.41. SPECT images (**a** and **e**) were reconstructed with postfiltering and represent the subjectively preferred image quality for reading. **a** represents our institutional standard for diagnostic imaging and reconstruction. All SPECT images have an identical window level and width

**Fig. 4:**
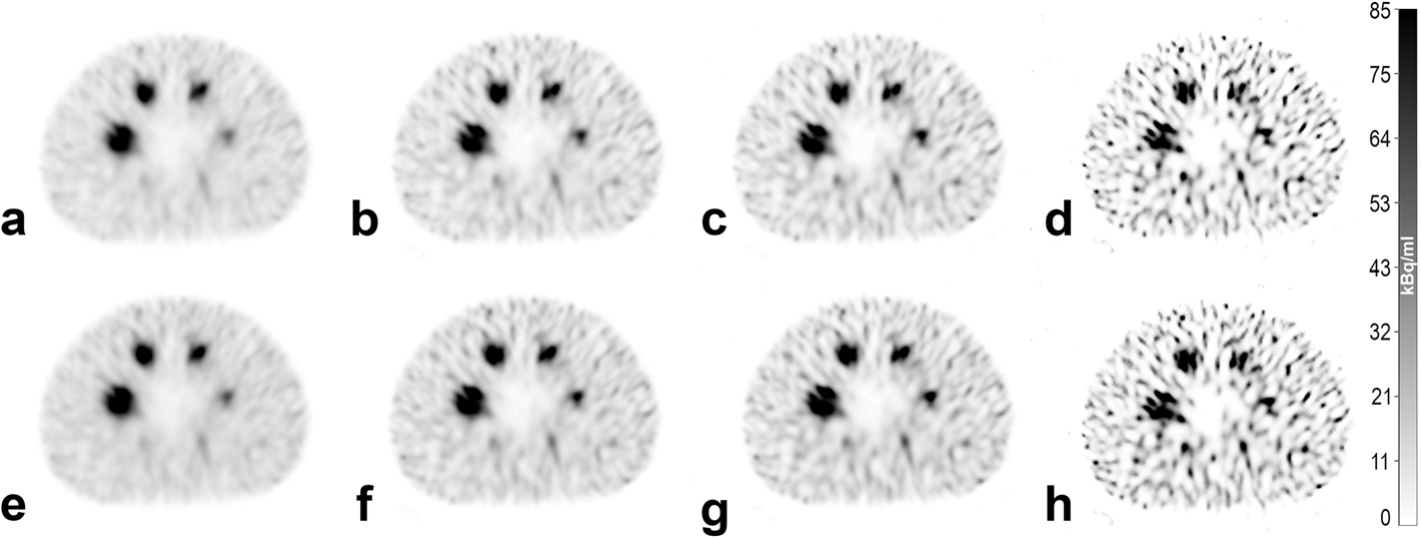
SPECT images reconstructed from data acquired with the NEMA acquisition protocol. Reconstruction was performed with 2i/10s (**a** and **e**), 4i/10s (**b** and **f**), 5i/15s (**c** and **g**) and 24i/10s (**d** and **h**). Scatter correction was performed with (**a**–**d**) SCF = 1.10 and (**e**–**h**) SCF = 0.41. **a** and **e** Reconstructed with postfiltering typically used in diagnostic imaging. All SPECT images have an identical window level and width

General image quality of the quantitative reconstructions for the spheres and the background is shown in Fig. 3 for the clinical acquisition protocol and Fig. 4 for the NEMA acquisition protocol. Four of the six largest spheres in the phantom were clearly visible as spheres for all examined reconstructions. The non-active lung insert also clearly differentiated from the background. The subjectively preferred image quality (MCK, HA, JW) is provided by the clinical acquisition protocol with 2i/10s and postfiltering (Fig. 3a and e). There is no preference for a specific SCF. Here, *N* ranges between 21% (Fig. 3e) and 25% (Fig. 3a) for the background.

The highest noise in the background was provided by the reconstructions with 24i/10s with SCF_1.10_ (Figs. 3/4d). Here, *N* ranges between 106% (Fig. 3d) and 116% (Fig. 4d). The results for all examined protocols and additional descriptive statistics are provided in the supplementary data (supplementary Tables 2 and 3).

## Discussion

A common clinically used qSPECT reconstruction algorithm was evaluated regarding quantitative accuracy and image quality using a standard phantom geometry. SPECT data acquired by two different acquisition protocols, a clinical protocol and a NEMA-oriented scan protocol, were reconstructed by iterative algorithms with different parametrizations (e.g., variations in the number of iterations and different factors for scatter correction). The two acquisition protocols were chosen to analyse the effect introduced by different numbers of projections for equivalent total examination times. Reconstructed data were evaluated regarding the effect of scatter correction and of the total number of iterations on the determined activity concentration. In addition, we compared the results of the quantitative reconstruction setups with a reconstruction setup used as a standard for diagnostic imaging in routine clinical practice. The phantom setup was chosen in concordance with activity concentrations typical for diagnostic workflows using ^99m^Tc-labelled radiopharmaceuticals.

Various setups for activity concentrations and activity ratios (e.g., sphere-to-background) are reported in the literature. Livieratos et al. [28] performed phantom measurements representing a bone scan–oriented setup with an activity concentration ratio of 4.8:1 (sphere = 240 kBq/ml and background = 50 kBq/ml). Collarino et al. [25] performed phantom measurements based on breast cancer imaging with four sets of activity concentration ratios and different background activity concentrations (e.g., sphere-to-background ratio of 9.7:1 and background = 3.6 kBq/ml). A showcase from this work reveals tumour-to-background ratios of 10:1 and 8:1 for the early (5 min p.i.) and delayed (90 min p.i.) acquisition. However, since our approach is not limited to a particular type of examination and the focus is primarily on quantitative analysis, we chose other activity concentrations. We assume that as long as the dead time is not exceeded, the investigated activity concentrations are of secondary importance compared with the object size with respect to the spatial resolution of the camera.

In our phantom setup, the default SCF_1.10_ in image reconstruction overestimates the portion of scattered photons within the photopeak window by a factor of 2.7. This result resulted in a significantly reduced AC_rec_ compared with the known AC in the phantom. Furthermore, qualitative parameters (e.g., SNR) were artificially affected because of the reduced count statistics. Thus, scatter correction with the object-optimized SCF_0.41_ resulted in an increased AC_rec_ closer to the real AC. Additionally, qualitative results were improved compared to results with default SCF_1.10_. However, both SCFs overestimate the known AC of the background. This finding has to be discussed regarding the methodology for estimating the SCF by using a small activity filled sphere in an inactive homogenous-filled water phantom (see supplementary information) [23].

Another objective for qSPECT was the parametrization of the quantitative reconstruction algorithm (e.g., number of iterations and subsets). We examined four different sets of iterations for the iterative OSEM algorithm. As known from PET imaging, with an increasing number of iterations, AC_rec_ increased and converged to the known AC [29]. However, a residual offset (underestimation of the AC) was observed. In parallel, image noise increased with an increasing number of iterations. On the other hand, the number of iterations showed no effect on the background AC. For a homogeneous, large object (e.g., the background VOI), it can be assumed that the reconstruction algorithm converges after a few iterations. It has to be hypothesized that the lowest examined number of total iterations (2i/10s) was sufficient to show convergence. Therefore, an effect introduced by the number of iterations on background AC was not observed.

Finally, the acquisition protocol (number of projections and duration of each projection) demonstrated a minor impact on the quantitative results for the spheres, in contrast to SCF or total number of iterations. Furthermore, there was no significant influence of the acquisition protocol on the estimated image quality parameters. In contrast, the acquisition protocol has an influence on the background. For the background, the best quantitative results were obtained with the clinical protocol, and the best qualitative results were obtained with the NEMA protocol. Using the NEMA protocol, the AC reconstructed in the background was overestimated compared with the known AC. At the same time, the standard deviations of the ACs are smaller with the NEMA protocol than with the clinical protocol, resulting in a reduction in image noise. Here, we concluded that the acquisition parameters, such as the number of projections and duration of projection time, are less relevant in volumes with high-count statistics (e.g., in the spheres) compared with regions with low-count statistics (e.g., in the background).

In summary, the best quantitative results were achieved with the object-specific SCF_0.41_ and a reconstruction set with 24 iterations and 10 subsets without postfiltering. In contrast, the best qualitative results were achieved with SCF_0.41_ with 2 iterations and 10 subsets and with postfiltering. In this parametrization, however, for diagnostic reading, the clinical acquisition protocol (regardless of SCF) was subjectively preferred.

In addition to the effects examined, further corrections have to be considered in qSPECT imaging (e.g., for photon attenuation, patient motion, dead time, radioactive decay and postfiltering of data) [4, 7–12]. These effects have not been analysed here.

In our study, photon attenuation was determined from low-dose CT data. Here, optimization of low-dose CT (e.g., the use of iterative reconstruction techniques) was demonstrated to be an innovative approach [30–32]. The influence/correction of dead time is not appropriate for the examined activity concentration typically observed in diagnostic applications but should be considered in the imaging of therapy nuclides (e.g., internal radionuclide therapy with ^177^Lu) using high activities [33].

One limitation in our reconstruction process is the use of a mean planar sensitivity value for the conversion of SPECT counts to an activity concentration. The software used only a single system sensitivity for both detector systems, even though the sensitivities of the single detectors slightly differed for the examined radionuclide. The sensitivity is determined only from planar imaging; thus, the sensitivity depends on the object-related properties (e.g., dimension and resulting scattering). A potential approach to overcome specific methodological limitations is the use of a three-dimensional sensitivity value by measuring a homogeneously filled phantom with known activity [9].

Furthermore, our approach is limited by the estimation of the object-specific SCF, which in turn depends on density composition, object shape, VOI volume and activity concentration [17]. Here, the reconstruction can perhaps be improved by Monte Carlo-based individual scatter correction [15, 34]. In the comparison between the Monte Carlo-based scatter correction and a correction with additional energy windows, Gils et al. [35] demonstrated for the NEMA phantom that the results of the reconstructed activity in the spheres and in the background differ by less than 10% for ^131^I. However, the portion of scattered photons within the photopeak of ^131^I may be different from ^99m^Tc, and an improvement may nevertheless be expected. Xiao et al. [36] showed for different phantoms that Monte Carlo-based scatter correction for ^99m^Tc in cardiac perfusion SPECT is superior to conventional window-based scatter correction. With identical noise levels, the contrast of the Monte Carlo-based scatter correction is 10 to 20% higher.

Furthermore, SPECT imaging and quantitative analysis are always influenced by other effects (e.g., resolution recovery). In our setup, only the four largest spheres (volume ≥ 2.5 ml) could be clearly separated from the background in the reconstructed SPECT data. However, segmentation was performed CT-based to prevent an additional bias by SPECT data-oriented delineation of contours. Furthermore, the whole analysis was performed for the largest sphere only to limit the influence of the partial volume effect on the primary analysis of HSRCs and SNR (e.g., caused by definitions of parameters, supplementary data showing results from all spheres). Nevertheless, depending on the spatial resolution of the imaging system, recovery correction is recommended [26].

In the future, the utilization of qSPECT imaging will become more important in routine clinical practice. The predicted use of this method is especially true for dosimetry in novel treatments (e.g., in radionuclide therapies) [5, 6]. Diagnostics will also benefit from qSPECT imaging, as it improves intraindividual comparability and even allows direct comparison of reconstructions of different SPECT/CT devices [37–40]. Further developments might focus on optimizing SPECT images for CT metal artefacts, such as those that occur for medical devices (e.g., postsurgery prosthesis management [41]).

Therefore, we recommend performing reconstruction for diagnostic imaging and auxiliary reconstruction optimized for quantitative analysis. From our phantom study, reconstruction with 24i/10s is the best choice for quantitative reconstruction.

## Conclusion

Quantitative SPECT imaging is feasible with the used (commercially available) reconstruction algorithm and hybrid SPECT/CT, and its consistent implementation in diagnostics may provide perspectives for quantification in routine clinical practice. When combining quantitative analysis and diagnostic imaging, we recommend using two different reconstruction protocols with task-specific optimized setups (quantitative vs qualitative reconstruction). Furthermore, individual scatter correction significantly improves both quantitative and qualitative results. Accepting factory settings may lead to errors and should always be critically questioned. The conversion of measured counts into quantitative measured quantities (Bq/ml) by the used quantitative factor leads to acceptable accuracies (less than 10% deviation from the true activity concentration) for spheres up to 11.5 ml in volume if appropriate image data acquisition and reconstruction are used.

## Supplementary Information

**Additional file 1.** Determination of the weighting factor for scatter correction (SCF)

**Additional file 2: Table S1** Effect of examined parameter on AC_rec_, HSRC and SNR. Results are exemplified for all sphere volumes in comparison to a reference protocol

**Additional file 3: Table S2** AC_rec_, HSRC and SNR from all sphere volumes of the phantom^a^ for all examined acquisition and reconstruction protocols

**Additional file 4: Table S3** AC_rec_ and noise from background^a^ for all examined acquisition and reconstruction protocols

## Data Availability

SPECT and CT raw datasets are available from the corresponding author on reasonable request. All data analysed during this study are included in this published article and its supplementary information files.

## Abbreviations

2i/10s: Two iterations and 10 subsets
4i/10s: Four iterations and 10 subsets
5i/15s: Five iterations and 15 subsets
24i/10s: Twenty-four iterations and 10 subsets
^99m^Tc: Technetium-99m
^177^Lu: Lutetium-177
AC: Activity concentration
CT: Computer tomography
FWHM: Full width at half maximum
HSRC: Hot spot recovery coefficient
LEHR: Low-energy, high resolution
N: Image noise
NEMA IEC: National Electrical Manufacturers Association International Electrotechnical Commission
OSEM: Ordered subset expectation maximization
PET: Positron emission tomography
qSPECT: Quantitative SPECT
SCF: Scatter weighting factor
SD: Standard deviation
SNR: Signal-to-noise ratio
SPECT: Single photon emission computed tomography
VOI: Volume of interest
