# Supplementary material for: Optimization of SPECT/CT imaging protocols for quantitative and qualitative 99mTc SPECT
Source: EJNMMI Phys. 2021 Jul 30;8:57. doi: 10.1186/s40658-021-00405-3 (PMC8324619; doi:10.1186/s40658-021-00405-3)
Supplement: Supplementary file 1 — Additional file 1. Determination of the weighting factor for scatter correction (SCF) [file 40658_2021_405_MOESM1_ESM.docx]

**Determination of the weighting factor for scatter correction (SCF).**

To determine the SCF for the IEC Phantom, the ^99m^Tc point source (volume = 0.52 ml, activity = 98.7 MBq) centrally positioned in the phantom was measured once with scattering medium (phantom with inserts and filled with water) and once without scattering medium (phantom without water and inserts). The optimal weighting factor can be calculated from the measured counts C in the energy windows and their energy window widths w:

$R= C_{pp}-k\cdot\frac{w_{pp}}{w_{sc}}\cdot C_{sc}$ (1)

with

$SCF=k\cdot\frac{w_{pp}}{w_{sc}}$ (2)

Each measured energy spectrum was normalized to its maximum. Then, the counts were summed in the energy window. To take into account the dependence of the energy spectrum on the projection angle (such as in the case of extended objects and patient tables), emissions were measured from four angular projections (0°, 90°, 180°, 270°). The mean value was calculated from the four projections.

The scan of the phantom without scattering medium yielded the true (unscattered) count sum R in the photopeak window (pp, 140.5 keV ± 10%, 126.45 - 154.55 keV, w_pp_ = 28.1 keV). The normalized count sums in the phantom without scattering medium are R (0°) = 13.657, R (90°) = 13.749, R (180°) = 13.562, and R (270°) = 13.966. The mean count sum of R is 13.733.

The scan of the phantom with scattering medium led to the count sums C_pp_ in the photopeak window (pp, 140.5 keV ± 10%, 126.45 - 154.55 keV, w_pp_ = 28.1 keV) and C_sc_ in the scatter window (sc, 120.0 keV ± 5%, 114.0 - 126.0 keV, w_sc_ = 12.0 keV). The normalized count sums in the phantom with scattering medium are C_pp_ (0°) = 15.374, C_pp_ (90°) = 16.312, C_pp_ (180°) = 15.309, C_pp_ (270°) = 16.701 and C_sc_ (0°) = 5.330, C_sc_ (90°) = 5.165, C_sc_ (180°) = 5.376, C_sc_ (270°) = 5.538. The means of C_pp_ and C_sc_ are 15.924 and 5.352, respectively.

According to Equations (1) and (2), the specific geometry provides coefficients of k = 0.175 (scatter multiplier) and SCF = 0.409.
