# Supplementary material for: Optimization of SPECT/CT imaging protocols for quantitative and qualitative 99mTc SPECT
Source: EJNMMI Phys. 2021 Jul 30;8:57. doi: 10.1186/s40658-021-00405-3 (PMC8324619; doi:10.1186/s40658-021-00405-3)
Supplement: Supplementary file 2 — Additional file 2: Table S1 Effect of examined parameter on ACrec, HSRC and SNR. Results are exemplified for all sphere volumes in comparison to a reference protocol [file 40658_2021_405_MOESM2_ESM.docx]

Supplementary Table 1. Effect of examined parameter on AC_rec_, HSRC and SNR. Results are exemplified for all sphere volumes in comparison to a reference protocol^a^.

| parameter of variation | ΔAC_rec_ | p^b^ | ΔHSRC | p^b^ | ΔSNR | p^b^ |
| --- | --- | --- | --- | --- | --- | --- |
|  | [kBq/ml] |  |  |  |  |  |
| V = 26.5 ml  SCF: 0.41 | +6.7 | < 0.0001 | +0.08 | < 0.0001 | +1.1 | n.s.^d^ |
| iteration set: 4i/10s | +4.1 | < 0.0001 | +0.05 | < 0.0001 | -8.2 | < 0.0001 |
| iteration set: 5i/15s | +5.6 | < 0.0001 | +0.07 | < 0.0001 | -11.9 | < 0.0001 |
| iteration set: 24i/10s | +6.6 | < 0.0001 | +0.08 | < 0.0001 | -17.9 | < 0.0001 |
| acquisition: NEMA^c^ | -0.9 | 0.04 | -0.01 | 0.04 | +0.6 | n.s.^d^ |
|  |  |  |  |  |  |  |
| V = 11.5 ml  SCF: 0.41 | +6.5 | < 0.0001 | +0.08 | < 0.0001 | +1.0 | 0.02 |
| iteration set: 4i/10s | +4.8 | < 0.0001 | +0.06 | < 0.0001 | -7.5 | < 0.0001 |
| iteration set: 5i/15s | +7.5 | < 0.0001 | +0.09 | < 0.0001 | -10.8 | < 0.0001 |
| iteration set: 24i/10s | +7.7 | < 0.0001 | +0.09 | < 0.0001 | -16.7 | < 0.0001 |
| acquisition: NEMA^c^ | -7.0 | < 0.0001 | -0.08 | < 0.0001 | +0.6 | n.s.^d^ |
|  |  |  |  |  |  |  |
| V = 5.6 ml  SCF: 0.41 | +5.4 | < 0.0001 | +0.06 | < 0.0001 | +0.9 | n.s.^d^ |
| iteration set: 4i/10s | +7.0 | < 0.0001 | +0.08 | < 0.0001 | -5.1 | < 0.0001 |
| iteration set: 5i/15s | +10.6 | < 0.0001 | +0.12 | < 0.0001 | -7.6 | < 0.0001 |
| iteration set: 24i/10s | +12.4 | < 0.0001 | +0.15 | < 0.0001 | -12.4 | < 0.0001 |
| acquisition: NEMA^c^ | -3.6 | 0.0006 | -0.04 | 0.0006 | +0.1 | n.s.^d^ |
|  |  |  |  |  |  |  |
| V = 2.6 ml  SCF: 0.41 | +6.3 | < 0.0001 | +0.07 | < 0.0001 | +1.0 | 0.07 |
| iteration set: 4i/10s | +11.7 | < 0.0001 | +0.14 | < 0.0001 | -1.4 | 0.07 |
| iteration set: 5i/15s | +17.4 | < 0.0001 | +0.20 | < 0.0001 | -2.7 | 0.0007 |
| iteration set: 24i/10s | +22.1 | < 0.0001 | +0.26 | < 0.0001 | -6.5 | < 0.0001 |
| acquisition: NEMA^c^ | -3.6 | 0.0003 | -0.04 | 0.0003 | -0.6 | n.s.^d^ |
|  |  |  |  |  |  |  |
| V = 1.1 ml  SCF: 0.41 | +3.1 | n.s.^d^ | +0.04 | n.s.^d^ | +0.3 | n.s.^d^ |
| iteration set: 4i/10s | +5.9 | 0.06 | +0.07 | 0.06 | -0.6 | 0.25 |
| iteration set: 5i/15s | +11.2 | 0.0009 | +0.13 | 0.0009 | -0.8 | 0.06 |
| iteration set: 24i/10s | +19.5 | < 0.0001 | +0.23 | < 0.0001 | -2.5 | < 0.0001 |
| acquisition: NEMA^c^ | +7.3 | 0.002 | +0.09 | 0.002 | +1.3 | 0.0002 |
|  |  |  |  |  |  |  |
| V = 0.5 ml  SCF: 0.41 | +4.7 | < 0.0001 | +0.05 | < 0.0001 | +0.6 | 0.01 |
| iteration set: 4i/10s | +2.2 | 0.15 | +0.03 | 0.15 | -0.2 | 1 |
| iteration set: 5i/15s | +3.0 | 0.03 | +0.04 | 0.03 | -0.5 | 0.19 |
| iteration set: 24i/10s | +7.6 | < 0.0001 | +0.09 | < 0.0001 | -0.8 | 0.009 |
| acquisition: NEMA^c^ | +2.2 | 0.03 | +0.03 | 0.03 | +0.5 | 0.04 |

^a^ clinical standard for diagnostic imaging and reconstruction (60 projections with 20 s/projection, SCF_1.10_, 2i/10s, Butterworth postfilter)

^b^ ANOVA post hoc test

^c^ NEMA acquisition protocol (120 projections and 10 s/projection)

^d^ not significant, tested by ANOVA

SCF - scatter weighting factor, AC_rec_ – mean reconstructed activity concentration, HSRC – hot spot recovery coefficient, SNR – signal-to-noise ratio
