# Supplementary material for: Optimization of SPECT/CT imaging protocols for quantitative and qualitative 99mTc SPECT
Source: EJNMMI Phys. 2021 Jul 30;8:57. doi: 10.1186/s40658-021-00405-3 (PMC8324619; doi:10.1186/s40658-021-00405-3)
Supplement: Supplementary file 3 — Additional file 3: Table S2 ACrec, HSRC and SNR from all sphere volumes of the phantoma for all examined acquisition and reconstruction protocols [file 40658_2021_405_MOESM3_ESM.docx]

Supplementary Table 2. AC_rec_, HSRC and SNR from all sphere volumes of the phantom^a^ for all examined acquisition and reconstruction protocols.

|  | SCF | iteration set | mean ± SD | median (IQR) | range | HSRC | SNR |
| --- | --- | --- | --- | --- | --- | --- | --- |
|  |  |  | [kBq/ml] | [kBq/ml] | [kBq/ml] |  |  |
| V = 26.5 ml | | | | | | | |
| clinical^b^ | 1.10 | 2i/10s^c^ | 67.8 ± 27.0 | 65.2 (48.1/86.3) | 25.6 - 133.5 | 0.797 | 22.1 |
|  |  | 4i/10s | 72.0 ± 29.2 | 71.6 (51.3/89.7) | 22.4 - 156.0 | 0.846 | 14.4 |
|  |  | 5i/15s | 72.7 ± 30.9 | 71.6 (51.7/89.7) | 18.2 - 163.4 | 0.854 | 10.3 |
|  |  | 24i/10s | 74.7 ± 38.3 | 70.5 (49.1/94.0) | 9.6 - 229.7 | 0.878 | 6.0 |
|  | 0.41 | 2i/10s^c^ | 74.7 ± 27.8 | 73.2 (54.5/94.0) | 31.0 - 130.3 | 0.878 | 23.7 |
|  |  | 4i/10s | 78.4 ± 29.6 | 79.0 (57.7/97.2) | 27.8 - 150.6 | 0.922 | 15.1 |
|  |  | 5i/15s | 79.0 ± 31.0 | 79.0 (57.7/97.2) | 22.4 - 163.4 | 0.929 | 10.8 |
|  |  | 24i/10s | 81.0 ± 37.7 | 78.0 (55.5/101.5) | 12.8 - 210.4 | 0.952 | 6.2 |
| NEMA^d^ | 1.10 | 2i/10s^c^ | 66.2 ± 26.5 | 63.8 (47.2/84.6) | 26.4 - 120.9 | 0.778 | 22.6 |
|  |  | 4i/10s | 70.5 ± 29.6 | 69.2 (49.5/88.9) | 22.0 - 147.3 | 0.829 | 14.7 |
|  |  | 5i/15s | 73.1 ± 32.5 | 70.3 (50.6/91.2) | 17.6 - 163.8 | 0.859 | 11.7 |
|  |  | 24i/10s | 72.8 ± 50.3 | 62.7 (37.4/96.7) | 4.4 - 300.1 | 0.856 | 4.6 |
|  | 0.41 | 2i/10s^c^ | 73.4 ± 28.0 | 70.3 (52.8/93.4) | 33.0 - 133.0 | 0.862 | 25.0 |
|  |  | 4i/10s | 77.5 ± 31.0 | 76.4 (56.1/96.7) | 26.4 - 162.7 | 0.911 | 16.3 |
|  |  | 5i/15s | 79.6 ± 33.5 | 78.0 (57.2/98.9) | 22.0 - 178.1 | 0.935 | 12.9 |
|  |  | 24i/10s | 80.0 ± 51.2 | 69.9 (44.0/104.4) | 5.5 - 318.8 | 0.940 | 5.1 |
| V = 11.5 ml | | | | | | | |
| clinical^b^ | 1.10 | 2i/10s^c^ | 66.8 ± 31.7 | 62.0 (42.7/86.5) | 20.3 - 147.4 | 0.785 | 21.7 |
|  |  | 4i/10s | 71.7 ± 36.5 | 68.4 (45.9/92.9) | 18.2 - 192.3 | 0.843 | 14.3 |
|  |  | 5i/15s | 76.0 ± 42.4 | 71.6 (46.1/97.2) | 15.0 - 243.6 | 0.894 | 10.9 |
|  |  | 24i/10s | 74.8 ± 54.6 | 64.1 (37.4/99.3) | 5.3 - 360.0 | 0.879 | 6.0 |
|  | 0.41 | 2i/10s^c^ | 73.4 ± 32.2 | 69.4 (49.1/92.9) | 27.8 - 151.7 | 0.863 | 23.2 |
|  |  | 4i/10s | 78.2 ± 37.0 | 74.8 (51.3/99.3) | 23.5 - 191.2 | 0.919 | 15.0 |
|  |  | 5i/15s | 82.7 ± 42.9 | 77.1 (53.2/106.8) | 19.2 - 236.1 | 0.971 | 11.4 |
|  |  | 24i/10s | 81.7 ± 54.5 | 70.5 (43.8/105.8) | 9.6 - 330.1 | 0.959 | 6.3 |
| NEMA^d^ | 1.10 | 2i/10s^c^ | 60.8 ± 29.5 | 55.0 (38.5/75.8) | 19.8 - 136.3 | 0.715 | 20.3 |
|  |  | 4i/10s | 65.7 ± 33.9 | 60.0 (40.9/83.5) | 14.3 - 155.0 | 0.772 | 13.5 |
|  |  | 5i/15s | 66.9 ± 34.8 | 61.6 (41.8/85.7) | 12.1 - 173.7 | 0.787 | 10.5 |
|  |  | 24i/10s | 68.1 ± 51.8 | 57.2 (31.9/90.1) | 1.1 - 312.2 | 0.801 | 4.3 |
|  | 0.41 | 2i/10s^c^ | 67.6 ± 31.2 | 61.6 (44.0/84.6) | 24.2 - 141.8 | 0.794 | 22.6 |
|  |  | 4i/10s | 72.2 ± 34.8 | 67.1 (47.3/91.2) | 18.7 - 160.5 | 0.848 | 15.0 |
|  |  | 5i/15s | 73.0 ± 35.2 | 68.2 (47.3/93.4) | 16.5 - 179.2 | 0.858 | 11.7 |
|  |  | 24i/10s | 74.6 ± 52.1 | 66.0 (38.0/96.7) | 2.2 - 318.8 | 0.876 | 4.7 |
| V = 5.6 ml | | | | | | | |
| clinical^b^ | 1.10 | 2i/10s^c^ | 52.7 ± 22.1 | 50.4 (36.7/65.2) | 20.3 - 100.4 | 0.620 | 16.3 |
|  |  | 4i/10s | 60.4 ± 29.1 | 57.6 (40.0/75.8) | 19.2 - 143.1 | 0.710 | 11.7 |
|  |  | 5i/15s | 64.8 ± 33.8 | 60.9 (42.1/80.1) | 15.0 - 177.3 | 0.761 | 9.0 |
|  |  | 24i/10s | 68.3 ± 47.0 | 59.8 (37.4/87.6) | 7.5 - 301.2 | 0.802 | 5.4 |
|  | 0.41 | 2i/10s^c^ | 59.0 ± 24.0 | 55.7 (42.6/73.7) | 23.5 - 107.9 | 0.693 | 17.7 |
|  |  | 4i/10s | 65.9 ± 30.0 | 63.0 (45.2/84.4) | 23.5 - 144.2 | 0.774 | 12.2 |
|  |  | 5i/15s | 69.6 ± 34.0 | 66.2 (46.4/86.5) | 21.4 - 175.2 | 0.818 | 9.3 |
|  |  | 24i/10s | 72.9 ± 46.6 | 67.3 (42.7/90.8) | 11.8 - 286.3 | 0.856 | 5.5 |
| NEMA^d^ | 1.10 | 2i/10s^c^ | 50.9 ± 22.7 | 48.1 (34.1/64.9) | 13.2 - 101.1 | 0.599 | 16.3 |
|  |  | 4i/10s | 57.9 ± 30.9 | 53.9 (35.0/76.9) | 8.8 - 136.3 | 0.681 | 11.6 |
|  |  | 5i/15s | 61.1 ± 36.7 | 56.1 (33.6/81.3) | 6.6 - 166.0 | 0.717 | 9.4 |
|  |  | 24i/10s | 61.5 ± 55.6 | 49.4 (23.6/86.1) | 0.0 - 289.1 | 0.722 | 3.7 |
|  | 0.41 | 2i/10s^c^ | 56.9 ± 25.6 | 52.8 (37.4/71.4) | 17.6 - 117.6 | 0.669 | 18.2 |
|  |  | 4i/10s | 63.6 ± 33.3 | 58.3 (38.0/82.8) | 13.2 - 158.3 | 0.747 | 12.8 |
|  |  | 5i/15s | 66.5 ± 38.8 | 60.9 (37.4/87.9) | 9.9 - 193.5 | 0.781 | 10.4 |
|  |  | 24i/10s | 66.8 ± 55.7 | 54.0 (28.0/90.1) | 1.1 - 348.4 | 0.785 | 4.1 |
| V = 2.6 ml | | | | | | | |
| clinical^b^ | 1.10 | 2i/10s^c^ | 38.2 ± 14.2 | 35.3 (26.7/46.1) | 18.2 - 68.4 | 0.449 | 10.7 |
|  |  | 4i/10s | 49.6 ± 25.5 | 43.7 (31.0/62.5) | 17.1 - 106.8 | 0.583 | 9.1 |
|  |  | 5i/15s | 53.6 ± 32.3 | 46.4 (29.9/69.8) | 13.9 - 126.0 | 0.630 | 7.2 |
|  |  | 24i/10s | 58.9 ± 47.6 | 46.1 (24.6/83.6) | 6.4 - 173.1 | 0.692 | 4.5 |
|  | 0.41 | 2i/10s^c^ | 44.1 ± 15.3 | 40.6 (31.8/52.3) | 23.5 - 75.8 | 0.518 | 12.1 |
|  |  | 4i/10s | 55.7 ± 26.4 | 50.2 (36.3/69.4) | 21.4 - 112.2 | 0.655 | 9.9 |
|  |  | 5i/15s | 59.7 ± 33.0 | 52.4 (36.0/76.9) | 18.2 - 131.4 | 0.702 | 7.7 |
|  |  | 24i/10s | 64.7 ± 46.1 | 55.0 (31.0/88.8) | 9.6 - 161.3 | 0.761 | 4.8 |
| NEMA^d^ | 1.10 | 2i/10s^c^ | 33.1 ± 9.6 | 31.9 (25.3/37.5) | 19.8 - 51.7 | 0.389 | 8.9 |
|  |  | 4i/10s | 44.4 ± 18.1 | 41.7 (30.8/52.8) | 17.6 - 83.5 | 0.522 | 8.2 |
|  |  | 5i/15s | 51.4 ± 24.9 | 47.3 (32.1/64.9) | 14.3 - 106.6 | 0.605 | 7.6 |
|  |  | 24i/10s | 55.7 ± 43.4 | 48.3 (23.9/78.0) | 1.1 - 153.9 | 0.655 | 3.3 |
|  | 0.41 | 2i/10s^c^ | 38.6 ± 11.6 | 36.4 (29.7/44.0) | 23.1 - 62.7 | 0.454 | 10.7 |
|  |  | 4i/10s | 51.0 ± 21.4 | 47.2 (35.2/61.6) | 22.0 - 100.0 | 0.599 | 9.6 |
|  |  | 5i/15s | 58.7 ± 29.1 | 52.8 (38.1/73.6) | 18.7 - 128.6 | 0.690 | 8.9 |
|  |  | 24i/10s | 62.9 ± 49.3 | 53.9 (28.6/83.5) | 1.1 - 206.6 | 0.739 | 3.8 |
| V = 1.1 ml | | | | | | | |
| clinical^b^ | 1.10 | 2i/10s^c^ | 22.2 ± 6.0 | 21.4 (15.9/25.6) | 12.8 - 29.9 | 0.261 | 4.5 |
|  |  | 4i/10s | 26.6 ± 9.8 | 25.6 (17.8/32.2) | 10.7 - 39.5 | 0.313 | 3.8 |
|  |  | 5i/15s | 30.6 ± 12.9 | 28.8 (19.7/37.2) | 9.6 - 48.1 | 0.360 | 3.3 |
|  |  | 24i/10s | 35.3 ± 18.6 | 29.9 (21.4/46.5) | 6.4 - 62.0 | 0.415 | 2.3 |
|  | 0.41 | 2i/10s^c^ | 24.7 ± 6.1 | 24.2 (17.2/28.5) | 13.9 - 32.0 | 0.290 | 4.7 |
|  |  | 4i/10s | 29.0 ± 9.7 | 27.8 (19.3/35.1) | 12.8 - 41.7 | 0.341 | 3.8 |
|  |  | 5i/15s | 33.2 ± 12.7 | 31.6 (21.5/40.6) | 12.8 - 50.2 | 0.390 | 3.4 |
|  |  | 24i/10s | 38.7 ± 18.1 | 36.9 (22.3/47.5) | 10.7 - 65.2 | 0.454 | 2.4 |
| NEMA^d^ | 1.10 | 2i/10s^c^ | 24.1 ± 4.6 | 23.1 (19.5/26.4) | 18.7 - 30.8 | 0.284 | 5.2 |
|  |  | 4i/10s | 31.2 ± 9.0 | 29.7 (24.7/34.1) | 20.9 - 45.1 | 0.367 | 4.9 |
|  |  | 5i/15s | 37.7 ± 13.8 | 36.2 (27.4/42.6) | 22.0 - 60.5 | 0.443 | 5.0 |
|  |  | 24i/10s | 49.5 ± 34.6 | 41.9 (26.2/62.5) | 13.2 - 131.9 | 0.581 | 2.8 |
|  | 0.41 | 2i/10s^c^ | 27.3 ± 4.9 | 26.4 (22.5/29.7) | 20.9 - 34.1 | 0.321 | 6.0 |
|  |  | 4i/10s | 35.0 ± 9.7 | 33.0 (27.6/37.7) | 24.2 - 50.6 | 0.411 | 5.6 |
|  |  | 5i/15s | 41.5 ± 14.7 | 39.6 (30.2/46.9) | 26.4 - 66.0 | 0.488 | 5.6 |
|  |  | 24i/10s | 52.7 ± 35.2 | 45.1 (27.5/66.1) | 15.4 - 134.1 | 0.619 | 3.0 |
| V = 0.5 ml | | | | | | | |
| clinical^b^ | 1.10 | 2i/10s^c^ | 13.1 ± 2.9 | 11.6 (8.5/13.9) | 9.6 - 17.1 | 0.153 | 1.0 |
|  |  | 4i/10s | 14.1 ± 4.5 | 11.6 (9.0/16.0) | 8.5 - 20.3 | 0.166 | 0.9 |
|  |  | 5i/15s | 15.5 ± 5.3 | 12.8 (9.8/18.2) | 9.6 - 22.4 | 0.182 | 0.8 |
|  |  | 24i/10s | 19.0 ± 10.5 | 14.6 (9.8/24.4) | 6.4 - 34.2 | 0.223 | 0.8 |
|  | 0.41 | 2i/10s^c^ | 17.3 ± 3.2 | 16.0 (11.3/19.2) | 12.8 - 21.4 | 0.204 | 1.9 |
|  |  | 4i/10s | 19.7 ± 5.5 | 17.1 (12.8/22.4) | 11.8 - 26.7 | 0.232 | 1.7 |
|  |  | 5i/15s | 21.8 ± 6.9 | 19.2 (13.9/25.4) | 11.8 - 29.9 | 0.256 | 1.5 |
|  |  | 24i/10s | 27.4 ± 13.9 | 23.0 (14.4/35.8) | 9.6 - 45.9 | 0.322 | 1.4 |
| NEMA^d^ | 1.10 | 2i/10s^c^ | 16.2 ± 3.7 | 14.3 (11.7/16.5) | 11.0 - 22.0 | 0.190 | 2.0 |
|  |  | 4i/10s | 18.6 ± 6.3 | 16.4 (12.1/19.8) | 11.0 - 27.5 | 0.218 | 1.8 |
|  |  | 5i/15s | 18.6 ± 8.2 | 16.3 (11.4/19.6) | 9.9 - 29.7 | 0.219 | 1.4 |
|  |  | 24i/10s | 22.9 ± 20.2 | 18.7 (9.5/24.2) | 4.4 - 55.0 | 0.269 | 0.9 |
|  | 0.41 | 2i/10s^c^ | 19.0 ± 3.3 | 17.6 (14.0/19.8) | 14.3 - 23.1 | 0.223 | 2.5 |
|  |  | 4i/10s | 21.9 ± 5.8 | 19.8 (15.6/22.9) | 14.3 - 29.7 | 0.258 | 2.3 |
|  |  | 5i/15s | 21.7 ± 7.1 | 18.7 (14.7/23.1) | 13.2 - 31.9 | 0.255 | 1.7 |
|  |  | 24i/10s | 26.5 ± 18.6 | 21.4 (13.7/29.1) | 8.8 - 58.3 | 0.312 | 1.0 |

^a^ known activity concentration in the spheres was 85.1 kBq/ml

^b^ acquisition with 60 projections with 20 s/projection,

^c^ with postfiltering (Butterworth, cut-off frequency = 0.5, power = 10)

^d^ acquisition with 120 projections with 10 s/projection,

SCF – scatter weighting factor, AC_rec_ – reconstructed activity concentration, SD – standard deviation, IQR – interquartile range, HSRC – hot spot recovery coefficient, SNR – signal-to-noise ratio
