# Supplementary material for: Optimization of SPECT/CT imaging protocols for quantitative and qualitative 99mTc SPECT
Source: EJNMMI Phys. 2021 Jul 30;8:57. doi: 10.1186/s40658-021-00405-3 (PMC8324619; doi:10.1186/s40658-021-00405-3)
Supplement: Supplementary file 4 — Additional file 4: Table S3 ACrec and noise from backgrounda for all examined acquisition and reconstruction protocols [file 40658_2021_405_MOESM4_ESM.docx]

Supplementary Table 3. AC_rec_ and noise from background^a^ for all examined acquisition and reconstruction protocols.

|  | SCF | iteration set | mean ± SD | median (IQR) | range | noise |
| --- | --- | --- | --- | --- | --- | --- |
|  |  |  | [kBq/ml] | [kBq/ml] | [kBq/ml] | [%] |
| clinical^b^ | 1.10 | 2i/10s^c^ | 10.5 ± 2.6 | 9.9 (8.5/11.8) | 4.3 - 20.3 | 24.7 |
|  |  | 4i/10s | 10.4 ± 4.3 | 9.6 (7.5/12.8) | 2.1 - 32.0 | 41.5 |
|  |  | 5i/15s | 10.5 ± 6.0 | 9.6 (6.4/12.8) | 1.1 - 44.9 | 57.0 |
|  |  | 24i/10s | 10.2 ± 10.8 | 7.5 (3.2/12.8) | 0.0 - 101.5 | 105.7 |
|  | 0.41 | 2i/10s^c^ | 12.3 ± 2.6 | 11.8 (10.7/13.9) | 6.4 - 21.4 | 21.3 |
|  |  | 4i/10s | 12.2 ± 4.4 | 11.8 (8.5/13.9) | 3.2 - 31.0 | 36.0 |
|  |  | 5i/15s | 12.4 ± 6.2 | 10.7 (8.2/15.0) | 2.1 - 50.2 | 49.7 |
|  |  | 24i/10s | 12.2 ± 11.0 | 9.6 (5.3/15.2) | 0.0 - 119.6 | 90.5 |
| NEMA^d^ | 1.10 | 2i/10s^c^ | 11.4 ± 2.4 | 11.0 (9.8/13.2) | 5.5 - 20.9 | 21.3 |
|  |  | 4i/10s | 11.4 ± 4.0 | 11.0 (8.8/13.2) | 3.3 - 29.7 | 35.1 |
|  |  | 5i/15s | 11.2 ± 5.3 | 9.9 (7.7/13.2) | 2.2 - 37.4 | 47.2 |
|  |  | 24i/10s | 11.5 ± 13.2 | 7.6 (3.3/14.3) | 0.0 - 103.3 | 115.9 |
|  | 0.41 | 2i/10s^c^ | 12.8 ± 2.4 | 12.1 (11.0/14.3) | 6.6 - 20.9 | 18.9 |
|  |  | 4i/10s | 12.9 ± 4.0 | 12.1 (9.9/15.4) | 4.4 - 29.7 | 30.8 |
|  |  | 5i/15s | 12.6 ± 5.2 | 12.1 (8.8/15.4) | 3.3 - 35.2 | 41.0 |
|  |  | 24i/10s | 12.9 ± 13.1 | 8.8 (4.4/16.7) | 0.0 - 100.0 | 101.3 |

^a^ known activity concentration of the background was 10.6 kBq/ml

^b^ acquisition with 60 projections with 20 s/projection,

^c^ with postfiltering (Butterworth, cut-off frequency = 0.5, power = 10)

^d^ acquisition with 120 projections with 10 s/projection,

SCF – scatter weighting factor, SD – standard deviation, IQR – interquartile range
